# AMP Deaminase 1 Gene Polymorphism and Heart Disease—A Genetic Association That Highlights New Treatment

**DOI:** 10.1007/s10557-013-6506-5

**Published:** 2014-01-16

**Authors:** Ryszard T. Smolenski, Iwona Rybakowska, Jacek Turyn, Paweł Romaszko, Magdalena Zabielska, Anne Taegtmeyer, Ewa M. Słomińska, Krystian K. Kaletha, Paul J. R. Barton

**Affiliations:** 1 Department of Biochemistry, Medical University of Gdansk, Debinki 1, 80-211 Gdansk, Poland; 2 Department of Biochemistry and Clinical Physiology, Medical University of Gdansk, Debinki 1, 80-211 Gdansk, Poland; 3 Department of Clinical Pharmacology and Toxicology, University Hospital, Zurich, Switzerland; 4 NIHR Cardiovascular Biomedical Research Unit, Royal Brompton and Harefield NHS Foundation Trust, Sydney Street, London, SW3 6NP UK

**Keywords:** AMP deaminase, Nucleotides, Ischemic heart disease, Heart failure, AMP regulated protein kinase, Genetic polymorphism

## Abstract

Nucleotide metabolism and signalling is directly linked to myocardial function. Therefore analysis how diversity of genes coding nucleotide metabolism related proteins affects clinical progress of heart disease could provide valuable information for development of new treatments. Several studies identified that polymorphism of AMP deaminase 1 gene (AMPD1), in particular the common C34T variant of this gene was found to benefit patients with heart failure and ischemic heart disease. However, these findings were inconsistent in subsequent studies. This prompted our detailed analysis of heart transplant recipients that revealed diverse effect: improved early postoperative cardiac function associated with C34T mutation in donors, but worse 1-year survival. Our other studies on the metabolic impact of AMPD1 C34T mutation revealed decrease in AMPD activity, increased production of adenosine and de-inhibition of AMP regulated protein kinase. Thus, genetic, clinical and biochemical studies revealed that while long term attenuation of AMPD activity could be deleterious, transient inhibition of AMPD activity before acute cardiac injury is protective. We suggest therefore that pharmacological inhibition of AMP deaminase before transient ischemic event such as during ischemic heart disease or cardiac surgery could provide therapeutic benefit.

## Genetic Analysis of AMP Deaminase Mutations in Humans and its Impact in Heart Disease

A genetic background to the diversity seen in the clinical progression of heart disease is well documented. Genetic variants that lead to halted or delayed disease progression are particularly interesting as they may provide a basis for new therapies. Genetic diversity in pathways involving nucleotide metabolism are particularly important due to the latter’s direct links to myocardial function and metabolic regulation [1]. Several polymorphisms of the AMP deaminase 1 (*AMPD1*) gene have been described [2]. The C34T (Glu12Stop) mutation in exon 2 is by far the most common in the general population with an allele frequency of 10–14 % [3]. Loh et al. [4] were the first to describe a benefit of the C34T mutation in patients with heart disease. This study conducted in a group of 132 patients with dilated cardiomyopathy demonstrated that the probability of surviving without transplantation for more than 5 years is 8.6 times greater in patients carrying the C34T allele. Anderson et al. [5] confirmed a protective effect in ischemic heart disease demonstrating prolonged survival associated with the C34T mutation in a prospective study in 450 patients. Another study by Gastmann et al. [6] conducted in a group of 90 patients with congestive heart failure demonstrated better prognosis in patients possessing the C34T *AMPD1* mutation. Analysis of a consecutive group of 390 patients with left ventricular dysfunction revealed better survival in C34T allele carrier patients within a subgroup with ischemic cardiac dysfunction [7]. Other independent studies demonstrated a beneficial effect of the C34T mutation on metabolic aspects related to the cardiovascular system such as a lower level of an inhibitor of plasminogen activator and soluble von Willebrand factor in patients with coronary heart disease [8]. In contrast, three studies have indicated a lack or even a deleterious effect of the C34T *AMPD1* mutation in patients with heart disease. A large population study conducted in 935 post myocardial infarction and 433 heart failure patients with long term follow-up indicated increased mortality associated with the C34T mutation within patients with a history of myocardial infarction [9]. A prospective study in 686 patients with stable congestive heart failure did not demonstrate any impact of the C34T polymorphism on tested clinical, biochemical, echocardiographic, radionuclide or exercise parameters [10]. Analysis of 161 patients undergoing coronary revascularisation for clinical parameters including heart failure and cardiac death revealed lack of any impact of the C34T mutation [11]. In case of C34T polymorphism, assessment of impact on cardiovascular system could be complicated because this mutation was found to exert deleterious effects on muscle performance [12]. An interesting observation was highlighted by Safranow et al. that analysed 97 patients with coronary artery disease and 104 patients with heart failure [2]. Frequency of diabetes and obesity was lower in subjects with C34T mutation (Table 1).

**Table 1:** Summary of clinical effects of C34T mutation of AMP deaminase in heart disease

Number of patients	Diagnosis	Effect of C34T mutation	Reference
132	dilated cardiomyopathy	improved survival	[4]
450	ischemic heart disease	improved survival	[5]
90	congestive heart failure	better prognosis	[6]
390	left ventricular dysfunction	better survival in ischemic cardiac dysfunction group	[7]
109	coronary artery disease	lower level of inflammation/thrombosis markers	[8]
1368	myocardial infarction or heart failure	increased mortality in subgroup with prior myocardial infarction	[9]
686	stable congestive heart failure	no effect	[10]
161	coronary revascularisation	no effect	[11]
32	heart transplantation	high frequency in donors with good cardiac function	[13, 14]
262	heart transplantation	lower need for postoperative inotropic support, worse 1 year survival	[15]
201	coronary artery disease or heart failure	lower prevalence of diabetes	[2]

Our own studies have focused on the effects of the C34T mutation in patients undergoing cardiac surgery, including transplantation. We demonstrated a remarkably high frequency of C34T mutation of AMPD1 in 22 cardiac donors with good cardiac function as compared to 10 donors with echocardiographically confirmed acute cardiac failure [13, 14]. Donors with healthy hearts had a significantly higher frequency of C34T mutation also compared to control population (*n* = 207). Our recent analysis of 262 cardiac donors and 190 heart transplant recipients [15] highlighted a potential explanation for the discrepancy of published results by indicating that the C34T mutation induces diverse effects. While a protective effect was demonstrated in donor hearts with the C34T mutation in that they required less inotropic support, recipients of C34T allele carrying organs had a poorer 1 year survival. It seems that C34T mutation is protective for donor organ function in the short term but deleterious in the long term possibly due to the highly immunogenic post transplant environment. Consistent with this report we found that among a group of 153 patients undergoing coronary bypass surgery with use of cardiopulmonary bypass C34T carriers were better protected from functional deterioration [16]. In a group of patients without heart failure and with advanced coronary artery disease postoperative ejection fraction was similar to preoperative in patients with the C34T mutation while it was significantly decreased in patients without this mutation.

The C34T mutation of *AMPD1* affects not only the heart system. Long before its cardiovascular associations were observed, the C34T mutation was identified as cause of skeletal myopathy [17, 18]. Homozygotes for this mutation could have complete loss of AMP deaminase activity in skeletal muscle. It has been described as one of the most common inherited metabolic defects, with an estimated allele frequency of 20 %. Clinical symptoms associated with this deficiency are highly variable. While many subjects are asymptomatic others suffer from early fatigue, cramps and/or myalgia. Inability to maintain energy equilibrium, depletion of the muscle nucleotide pool or insufficient supply of anaplerotic substrates for the Krebs cycle in the exercising skeletal muscle were proposed as the underlying mechanisms of this syndrome. A varying effect of this mutation is known to be caused by alternative splicing of the *AMPD1* gene involving the elimination of the C34T nonsense mutation in exon 2 and allowing production of functional enzyme [17, 19].

Taken together these data suggest that the C34T mutation of *AMPD1* has diverse effects on the cardiovascular system and on the function of the human organism as a whole. During acute cardiovascular incidents it is clearly beneficial, but in the long term and in highly immunogenic environments such as after transplantation, the C34T mutation may have deleterious effect. Discrepancies between different studies attempting to clarify the impact of this alteration on human longevity may be a consequence of a different interplay of beneficial and deleterious effects in specific clinical conditions and under specific treatment. Besides basic information these studies have provided a clear indication when and how a potential therapy based on AMPD inhibition could be applied clinically.

## AMP Deaminase Isoforms and its Expression Pattern in the Heart

AMP-deaminase (AMPD) exists in human tissues in several isoforms with different kinetic properties, molecular weights and structures. These isoforms are the products of three different genes: *AMPD1*, *AMPD2* and *AMPD3*. In humans *AMPD1* is predominantly expressed in skeletal muscle, *AMPD2* is predominantly expressed in the brain, liver and heart and *AMPD3* is expressed mainly in the erythrocytes. In rodents the expression pattern of these three isoforms is different. While *AMPD1* is still the dominant skeletal muscle isoform and *AMPD2* is present in the liver, *AMPD3* is almost exclusively expressed in the heart [20]. This indicates that altered expression of *AMPD1* (as conferred by the C34T mutation) would not have any effect on AMPD expression in the mouse or rat heart. In humans the situation is very complex. While *AMPD2* is the main form expressed in human myocardium, transcripts for *AMPD3* and *AMPD1* are also present (our unpublished observations). Therefore the AMPD1 C34T mutation has an impact not only on skeletal muscle AMPD activity but also on its activity in myocardium where it causes a substantial reduction in enzyme activity even among heterozygotes [21, 22].

## AMP Deaminase Metabolic Function

AMP deaminase is an enzyme involved in the breakdown of nucleotides. It has several unique cellular functions and its activity and expression pattern are highly tissue specific. It plays a particularly important role in the breakdown of nucleotides in skeletal muscle. AMP deaminase forms part of the purine nucleotide cycle, which is designed to preserve adenylate’s energy charge and phosphorylation potential under conditions of insufficient energy supply. This cycle plays a crucial role in regulating the adenine nucleotide pool, in the synthesis of guanine nucleotides and in the provision of anaplerotic substrates for the Krebs cycle. These processes are important in skeletal muscle so the activity of AMP deaminase is 30–100 times higher than in the other organs. Under conditions of heavy exercise, when AMP accumulates, AMP deaminase allows rapid breakdown of AMP to IMP allowing a higher value of phosphorylation potential and free energy from ATP hydrolysis that translates directly into improved exercise capacity to be maintained. After exercise, when energy use is decreased, the IMP (a polar molecule) that remained inside the cell is reincorporated back into the adenine nucleotide pool via the adenylosuccinate synthetase and adenylosuccinate lyase reactions. The latter reaction also releases fumarate that supports the operation of the Krebs cycle.

In the heart and non-muscle organs AMP deaminase is not involved in the purine nucleotide cycle, but plays a role in regulation of the adenine nucleotide pool and in the synthesis of guanine nucleotides. The contribution of the AMP deamination pathway to the overall catabolism of nucleotides seems to be lower in human cardiomyocytes than in rat cardiomyocytes [23, 24], but still accounts for 30 % of the total breakdown capacity.

## Increased Production of Adenosine due to AMP Deaminase Deficiency

An important consequence of AMP deaminase deficiency is an increased flux of substrates through the 5′-nucleotidase pathway and increased adenosine production under conditions of heavy exercise. Adenosine content was found to increase 14 times in skeletal muscle biopsies taken from patients with AMPD deficiency during exercise compared with a 2 fold increase in normal subjects [25, 26]. The physiological effects of increased adenosine production in this setting have not been evaluated. However, previous studies have shown that increased production of adenosine may ameliorate a number of pathological processes including those involved in heart failure [27]. Adenosine has long been recognised to increase coronary flow and to be involved in the autoregulatory loop between contractile cells and blood supply [28] and was classified as an autacoid, retaliating against external stimuli, which deplete intracellular ATP in the heart [29]. A number of other cardioprotective physiological effects of adenosine have been described, such as the antagonism of catecholamine mediated hypercontraction, an anti-aggregatory activity, the inhibition of adhesion and toxic free radical generation by polymorphonuclear leukocytes, the promotion of angiogenesis and the induction of preconditioning [30–34]. Adenosine inhibits T lymphocyte function both at the stage of blastic transformation and cytolysis [35–40]. Increased adenosine concentration is partially responsible for severe combined immunodeficiency syndrome observed in patients with inherited adenosine deaminase deficiency. These immunosuppressive effects are of particular interest during transplantation. Adenosine also affects several processes involved in the pathogenic mechanisms of heart failure such as TNFα and IL-6 production [41–44], apoptosis [45] and proliferation of fibroblasts and smooth muscle cells [46–50]. We have previously shown the significant capacity of human endothelial cells to degrade AMP via the deamination pathway [51]. An increased adenosine production in cardiac endothelial cells due to deficiency of AMPD is one possible mechanism of the cardioprotective effects. However, even changes in remote organs may equally exert beneficial cardiac effects. Preconditioning at a distance has been demonstrated, indicating that an increase in adenosine production outside the heart in skeletal muscle for example may exert beneficial cardiac effects [52]. It has been shown also that a period of brief myocardial ischemia and increased cardiac adenosine production are capable of attenuating platelet aggregation in remote arteries [53]. Treatment with A2 adenosine receptor agonists was found to exert beneficial effects in animal models of both acute and chronic heart failure [54].

Despite numerous reports indicating benefits of elevated adenosine in cardiovascular disease, caution is needed as some studies demonstrated deleterious effects. The AMISTAD trial reported reduced infarct size but increased number of adverse clinical events, including death, in patients infused with adenosine as an adjunct to thrombolysis [55]. The administration of adenosine could be proarrhythmic [56] or produce coronary artery steal phenomenon in patients with critical stenosis [57]. Besides cardiovascular effects adenosine can contribute to fluid-retaining disorders via its A1 receptor mediated effects in the kidney [58] or induce bronchospasm in some patients [59]. Adenosine effects on immune cell function mediated by purinergic receptors may also promote cancer progression [60]. While these deleterious effects are restricted to specific clinical scenarios, its careful monitoring is needed in any adenosine related therapy including potential treatment with AMPD inhibitors.

## Activation of AMP Regulated Protein Kinase by AMP Deaminase Inhibition

AMP is not only a substrate for the AMPD reaction but also an allosteric regulator that signals energy deficiency in the cell. AMP directly activates glycolytic enzymes and indirectly controls a broad range of cellular functions via AMP regulated protein kinase (AMPK). AMPK is a heterotrimeric protein that was initially described as a regulator of energy metabolism [61, 62] but later found to play a role in cytoprotection, cell growth and regeneration [63]. The metabolic role of AMPK includes activation of fatty acid transport into the mitochondria—accomplished by phosphorylation and inhibition of acetyl CoA carboxylase—and reduction of malonyl-CoA concentration. AMPK activates glucose transport into the cell by translocation of GLUT-4 into the membrane and controls the expression of enzymes of energy metabolism by phosphorylation of HIF-1. Besides its metabolic effects, AMPK is linked with the Akt kinase pathway and induction of proteins involved in cytoprotection and regeneration. Activation of AMPK in heart infarction or heart failure is currently considered as a therapeutic target [64]. AMPK is also believed to be activated by commonly used drugs such as metformin [65]. However, AMPK’s protective effects could be dependent on how and when it is activated [66]. Loss of regulatory feedback in AMPK due to mutation in its γ2 subunit (PRKAG2) resulting in constitutively increased activity is known to cause myocardial glycogen storage disease and cardiomyopathy [67–69]. Our current hypothesis is that inhibition of AMPD will act to amplify the AMP accumulation in cells with disrupted energy metabolism. This indirect AMPK activation by its endogenous activator could be superior to direct activation of AMPK (e.g. by AICAr) since AMPK activation would be restricted to cells and conditions where this activation is necessary. We have in vitro evidence showing that high AMPD activity could suppress AMPK activity, possibly by competition for AMP. Recent study on the mechanism of AMPK activation by metformin has suggested involvement of inhibition of AMPD [70].

## Inhibition of AMP Deaminase as a Therapeutic Strategy

We have developed several possible therapeutic strategies based on the beneficial effects of adenosine that are applicable during cardiac surgery. We have established that adenosine administration both as a constituent of cardioplegic solution or as an infusion following cardioplegic arrest is beneficial [71, 72]. Administration of adenosine at the time of cardioplegic arrest, after reperfusion or after myocardial infarction is undergoing clinical evaluation. We have also developed a procedure that allows endogenous adenosine production in normoxic cardiac cells to increase by combined application of adenosine metabolism inhibitors and substrates for nucleotide synthesis [73]. We have shown that application of this procedure following experimental transplantation resulted in an improvement in all aspects of cardiac mechanical function, roughly a threefold decrease in postischemic cardiac neutrophil infiltration and an increase in myocardial ATP concentration. The discovery that genetic alterations of nucleotide metabolism that potentially leads to enhanced adenosine production and result in improved clinical outcome in heart dysfunction indicates new areas where such treatment could be applied.

The limited availability of specific inhibitors of AMPD is a major drawback for testing the regulation of AMPD in heart disease. Such inhibitors are not commercially available, although a procedure for chemical synthesis has been described [74] and preliminary evaluation of their effects has been performed as part of a PhD thesis [75]. We have followed this procedure and chemically synthesized a most effective compound: 3-[2-(3-carboxy-4-bromo-5,6,7,8-tetrahydronaphthyl)ethyl]-3,6,7,8-tetrahydroimidazo[4,5-*d*][1, 3]diazepin-8-ol (AMPDI). We have performed several preliminary studies using AMPDI with isolated AMPD, with heart homogenates, in isolated rat cardiomyocytes, perfused hearts and in mice in vivo [76–78]. In each case the efficiency of AMPD inhibition was confirmed, although in cardiomyocytes, perfused hearts and in vivo the concentration of inhibitor had to be several orders of magnitude higher than for isolated enzyme or heart homogenate alone. We have established that even at these high concentrations AMPDI remained specific for AMPD inhibition. While further studies are necessary to clarify the difference in effective concentration we believe that we have optimized the conditions for in vivo use of AMPDI. We have conducted a preliminary assessment of AMPDI effects in a mouse model of cardiac hypoxia and have demonstrated a protective effect [78]. We have established that the half life of AMPDI in mouse blood is relatively short (about 30 min) and that AMPD inhibition in vivo is transient, even with continuous infusion. AMPDI could therefore be a good tool to study the acute effects of AMPD inhibition, but the design of chemicals suitable for long term in vivo use and as drug candidates requires further work.
